# Genetically Engineered Mouse Models for Liver Cancer

**DOI:** 10.3390/cancers12010014

**Published:** 2019-12-19

**Authors:** Kyungjoo Cho, Simon Weonsang Ro, Sang Hyun Seo, Youjin Jeon, Hyuk Moon, Do Young Kim, Seung Up Kim

**Affiliations:** 1 Yonsei Liver Center, Yonsei University College of Medicine, Seoul 03722, Korea; kyungjoo89@yuhs.ac (K.C.); SIMONR@yuhs.ac (S.W.R.); SSHING94@yuhs.ac (S.H.S.); HMOON@yuhs.ac (H.M.); 2 Brain Korea 21 PLUS Project for Medical Science College of Medicine, Yonsei University, Seoul 03722, Korea; 3 Institute of Gastroenterology, Yonsei University College of Medicine, Seoul 03722, Korea; 4 Department of Life Science, Sahmyook University, Seoul 03722, Korea; YOUJIN0770@yuhs.ac; 5 Department of Internal Medicine, Yonsei University College of Medicine, Seoul 03722, Korea

**Keywords:** hepatocellular carcinoma, genetically engineered mouse, hydrodynamics-based transfection, sleeping beauty transposon, CRISPR/Cas9

## Abstract

Liver cancer is the fourth leading cause of cancer-related death globally, accounting for approximately 800,000 deaths annually. Hepatocellular carcinoma (HCC) is the most common type of liver cancer, comprising approximately 80% of cases. Murine models of HCC, such as chemically-induced models, xenograft models, and genetically engineered mouse (GEM) models, are valuable tools to reproduce human HCC biopathology and biochemistry. These models can be used to identify potential biomarkers, evaluate potential novel therapeutic drugs in pre-clinical trials, and develop molecular target therapies. Considering molecular target therapies, a novel approach has been developed to create genetically engineered murine models for HCC, employing hydrodynamics-based transfection (HT). The HT method, coupled with the Sleeping Beauty transposon system or the CRISPR/Cas9 genome editing tool, has been used to rapidly and cost-effectively produce a variety of HCC models containing diverse oncogenes or inactivated tumor suppressor genes. The versatility of these models is expected to broaden our knowledge of the genetic mechanisms underlying human hepatocarcinogenesis, allowing the study of premalignant and malignant liver lesions and the evaluation of new therapeutic strategies. Here, we review recent advances in GEM models of HCC with an emphasis on new technologies.

## 1. Introduction

Liver cancer is a major health concern worldwide; it is the second leading cause of cancer-related deaths in East Asia and sub-Saharan Africa and the sixth in Western countries [1,2]. The main risk factors for liver cancer are well known and include hepatitis B virus (HBV) infection, hepatitis C virus (HCV) infection, alcohol intake, liver cirrhosis, metabolic syndrome, or a combination of the above [3]. Hepatocellular carcinoma (HCC) develops through a series of genetic and epigenetic changes in proto-oncogenes and tumor suppressor genes in the liver environment, wherein hepatic fibrosis or cirrhosis occurs after sustained liver damage [4].

Therapeutic options for patients with chronic liver disease and cirrhosis exist, such as potent antiviral therapies for hepatitis B and C, alcohol abstinence programs, and exercise routines for metabolic syndrome. However, HCC patients who are diagnosed at an advanced stage have limited medical options; moreover, these may only increase the mean overall survival by a few months [5]. Furthermore, the process of anti-HCC drug discovery has been difficult and inefficient, as reflected by the high attrition rate of drugs that enter preclinical testing but fail to attain FDA (Food and Drug Administration) approval [6]. Therefore, understanding the molecular signaling pathways or protein–protein interactions that play crucial roles in hepatocarcinogenesis should provide important information for the treatment of HCC.

The mouse (*Mus musculus*) is considered the best animal model for cancer research due to its physiological and molecular similarities with human biology, in addition to its advantages in terms of size, reproductive capacity, and lifespan. Mouse models for HCC have been developed through the introduction of genetic changes that contribute to the pathogenesis of the disease; however, with regard to the modeling of spontaneous HCC arising from a chronic inflammatory environment, there are considerable challenges that need to be overcome. Moreover, as it is becoming increasingly clear that there is no unique molecular pathway underlying the pathogenesis of HCC, various models are needed to mimic the different types of liver tumorigenesis.

This review aimed to provide a blueprint to understand the pathogenesis of HCC and optimize the preclinical models used in drug efficacy testing.

## 2. Non-Genetically Engineered Mouse (GEM) Models

### 2.1. Chemically Induced Models

Many carcinogens that induce HCC have been identified. It has been reported that both synthetic chemicals (such as carbon tetrachloride (CCl_4_), diethylnitrosamine (DENA), 2-acetylaminofluorene (AAF), N-nitrosodimethylamine, arsenic, o-aminoazotoluene, N-nitrosomorpholine (NMOR), and 1,2-dichloroethane) and natural substances (such as xanthosine, pyrrolizidine alkaloids, and safrole) can be liver carcinogens [7,8]. These carcinogens are administered to the animal through food, drinking water, gas inhalation, or intraperitoneal or subcutaneous injections. Chemically induced HCC models are sometimes combined with liver resection to induce cell proliferation, which increases the frequency of mutations induced by the chemical. The characteristics of each carcinogenic chemical are summarized in Table 1.

Hepatic cirrhosis and multifocal HCC developed 50 weeks after intraperitoneal injection of DENA [9,10,11]. When using NMOR as a carcinogen, HCC and lung metastasis were observed after approximately 12 weeks of carcinogen supply in drinking water at a concentration of 120 ppm [12]. A choline-deficient diet with a low intake of methionine over 2 weeks can lead to rapid death of liver cells, and their combination with DENA, azaserine, or AAF treatments can accelerate the development of liver cancer cells [8,13,14,15,16,17]. This model, however, has the limitation of a large variation in the susceptibility to choline deficiency.

Metastasis can be induced by treatment with carcinogens; however, the low long-term survival rate of the treated animals makes it difficult to assess metastasis. In 2005, Yoshino et al. established an animal metastasis model that showed a significantly higher survival rate than previous models [18]. It was developed by treatment with DENA at a concentration of 120 ppm, followed by the supply of water containing NMOR at a low concentration (40 or 80 ppm) for 14 weeks. The 40 ppm NMOR-treated model is considered to be suitable to study the mechanism of metastasis. Here, HCC develops without lung metastasis until 22 weeks and frequent lung metastasis is found after 40 weeks.

### 2.2. Transplantation Models

Since 1969, when it was first identified that tumor cells implanted subcutaneously into immunodeficient mice gave rise to cancer, many types of cell lines and tissues have been implanted in mice for cancer research [19,20]. The transplantation model provides a suitable niche for the survival of tumor cells in vivo. Transplantation models that use human tumor sources are classified by the type of sample (tumor cell culture or tumor tissue obtained from surgery) and the anatomical location of transplantation (ectopic or orthotopic) [21]. The ectopic xenograft model has the advantage of allowing easy measurement of tumor size, thus facilitating antitumor drug efficacy testing in vivo [22]. For the exploration of immunotherapies for HCC, syngeneic models established with immunocompetent mouse strains are preferable; such models, however, cannot recapitulate the histology, natural carcinogenesis characteristics, or microenvironment of human HCC [23,24,25]. In addition, a vaccination effect may occur due to differences in the human and mouse immune systems [26]. Humanized mouse models established with patient-derived xenografts and human peripheral-blood mononuclear cells (PBMCs) can recapitulate human HCC and the human immune system, although they are expensive and require long periods of time [27].

Ma et al. isolated CD133-expressing HCC cells from human cell lines and used them for transplantation into immunodeficient mice to test their characteristics and resistance to chemical drugs. These CD133-expressing tumor cells survived at a higher rate than those that did not express CD133 [28,29]. Researchers at the University Hospital Bonn injected alcohol and thioacetamide into C3H mice to induce liver fibrosis and, then, transplanted Hepa129 cells into the liver [30], showing that tumor growth in the fibrotic liver is faster than that in the normal liver. In addition, Kornek et al. found that the expression levels of vascular endothelial growth factor (VEGF), vascular endothelial growth factor receptor (VEGFR), and matrix metalloproteinase-2 (MMP-2) and MMP-9 were higher in tumors derived from fibrotic livers. This xenograft model has been a useful tool in drug efficacy testing in the context of liver fibrosis. The various transplantation models are summarized in Table 2.

## 3. GEM Models

### 3.1. Traditional GEM Model

The best model for human cancer should resemble the pathological and molecular characteristics of human malignancy [31]. The model should also allow the investigation of the interactions between the tumor microenvironment (TME) and tumor cells and provide information on the molecular signaling pathways leading to cancer [32]. Transgenic mice were first created in the 1980s and enabled the study and characterization of molecular signaling pathways in human malignant tumors [33,34,35].

Transgenic models have been engineered to express oncogenes or inactivate tumor suppressor genes; when performed in the liver, these genetic manipulations have been shown to induce HCC [31,32]. Such gene expression can be restricted to liver cells using liver-specific promoters such as the albumin promoter. Genes that are used to develop GEM models for HCC include those related to the hepatitis virus, cell proliferation, and apoptosis.

### 3.2. Viral Genes

Chronic viral hepatitis is the most common etiology of HCC and accounts for 80% of all HCC worldwide [36]. Hepatitis B is an endemic disease in China, Southeast Asia, and Sub-Saharan Africa, where liver cancer shows a high incidence rate. HCV is more widely distributed in the United States and Europe than HBV or the human immunodeficiency virus (HIV) [37].

#### 3.2.1. Hepatitis B Virus (HBV)

HBV, a circular DNA virus that has four open reading frames, is difficult to propagate in vitro using cell culture. The HBV X protein (HBx) is commonly used to induce HCC in murine models. In 1994, Koike et al. showed that HCC developed in transgenic mice with high HBx expression within 13 to 24 months in 84% of cases [38]. DNA analyses of these mice revealed that persistent HBx expression induced DNA synthesis and secondary mutations in many hepatocytes. Moreover, HBx transgenic mice showed differences in hepatocarcinogenesis depending on the HBV genotypes. HCC occurred after the expression of HBx from HBV genotype C in transgenic mice, whereas HBx of other genotypes hardly induced HCC [39].

In addition to HBx, Chisari et al. developed a transgenic model overexpressing large envelope polypeptides of HBV [40]. This model demonstrated that the expression of a single structural viral gene is sufficient to induce malignant transformation. The hepatocyte injury due to the accumulation of a viral product led to HCC via an inflammatory response, regeneration, transcriptional deregulation, and aneuploidy. This model supported the hypothesis that sustained hepatocyte injury can induce secondary genetic events that cause unrestricted proliferation [41].

#### 3.2.2. Hepatitis C Virus (HCV)

HCV is an RNA virus that is not integrated into the host genome but bears proteins that exert a wide range of biological effects by interacting with many host cell factors [42]. Several models have been developed to understand the tumorigenesis of HCC by HCV.

Experiments using cell culture systems have shown that the core protein of HCV itself can regulate various cell functions, which may be directly associated with the development of HCV-related HCC [43]. The HCV core protein cooperates with the *Hras* oncogene in rat embryo fibroblasts, inhibits apoptosis associated with *c-Myc*, and represses transcription of *Tp53* [44,45]. Moreover, it allows the peroxisome proliferator-activated receptor (PPAR) to interact with various proteins leading to hepatic carcinogenesis [46,47,48].

To determine the contribution of HCV structural proteins to hepatocarcinogenesis, Kamegaya et al. developed transgenic mice expressing only the HCV core protein or both the HCV core protein and E1/E2 proteins [49]. HCC cell proliferation was not significantly different between the two groups; however, HCCs in HCV core-E1/E2 transgenic mice showed significantly lower cell death rates than those in HCV core transgenic mice. In addition, tumor size in HCV core-E1/E2 transgenic mice was larger than that in HCV core transgenic mice.

#### 3.2.3. Woodchuck Hepatitis Virus (WHV)

WHV belongs to the hepadnavirus family. In woodchucks, it induces hepatitis and hepatocellular carcinoma that are remarkably similar to those associated with HBV infection in humans. Ultimately, almost all WHV infections develop into well-differentiated HCCs. HCC is generally caused by the overexpression of the *c-Myc* and *N-Myc* genes when the WHV DNA is integrated at a specific location of the genome [50,51].

Viral-derived HCC models are valuable for long-term testing of chemoprevention strategies and evaluating targeted therapies for established HCCs. However, these models have severe limitations, such as a long latency of HCC development (usually over 2 years).

### 3.3. Manipulation of Host Proto-Oncogenes and Tumor Suppressor Genes

To induce HCC, liver-specific promoters are employed to drive the expression of oncogenes. Several promoters have been used for hepatic expression, such as albumin, metallothionein, transtthyretin, and liver activator protein (LAP) [49,52,53,54,55].

Sandgren et al. developed transgenic mice that specifically expressed *c-Myc* in the liver using an albumin enhancer/promoter. In this experiment, *c-Myc* expression resulted in mild to severe levels of hepatic dysfunction in young mice and hepatoblastoma in old mice after 15 months [56]. Furthermore, it was shown that *c-Myc* could lead to a mutation in the *β-catenin* gene that led to changes in *β-catenin* signaling transduction, which eventually led to HCC [57].

To investigate the interaction between *c-Myc* and transforming growth factor alpha (*TGF-α*) in HCC development, a double transgenic mouse model was developed. It expressed *c-Myc* through an albumin enhancer/promoter and *TGF-α* through a metallothionein 1 promoter [58,59,60,61]. This model significantly reduced the HCC onset time compared to transgenic mice expressing *c-Myc* or *TGF-α* individually. The mouse model co-expressing *c-Myc* and *TGF-α* induced continuous hepatocyte proliferation, followed by tumor development 2 months later [59]. Compared to lesions caused by *c-Myc* expression alone at 10 weeks, the simultaneous expression of *c-Myc* and *TGF-α* significantly increased the production of reactive oxygen species (ROS), genetic instability, and loss of heterozygosity [62]. Other double transgenic mice models expressing *c-Myc* plus E2F1 or *c-Myc* plus EGF were also developed [61,63,64].

β-Catenin, the key downstream effector of the Wnt signaling pathway, plays an important role in the liver. Activation of the Wnt/β-catenin pathway in humans can occur via an activating mutation within the *β-catenin* gene or the reduced expression of adenomatous polyposis coli (*APC*), a negative regulator of β-catenin [57]. GEM models expressing an activated form of β-catenin or with a liver-specific *Apc* knockout showed hepatomegaly or HCC with a long latency [65]. However, the coexpression of activated β-catenin with an activated *Ras* led to HCC as early as 8 weeks [66].

The phosphatase and tensin homolog (*PTEN*) is a tumor suppressor that negatively regulates the *PI3K-Akt* signaling pathway, which, in turn, regulates cell survival, proliferation, and energy metabolism. GEM models with a *Pten* deletion in the liver exhibited HCC after 44 weeks [67]. The characteristics of each mouse model using proto-oncogenes or tumor suppressor genes are summarized in Table 3.

### 3.4. Inducible Gene Expression Models

Employing a liver-specific promoter allows an oncogene to be specifically expressed in cells of the hepatic lineage from embryogenesis [32]. Considering HCC usually develops in adults via somatic mutations, the expression of an oncogene since early embryonic development might cause embryonic lethality or unexpected abnormal characteristics that could make the HCC model deviate from human HCC [68]. Additionally, to investigate the so-called oncogenic addiction, one might want to inactivate the driver oncogene in transgenic mice after establishing liver cancer. Thus, systems that exert temporal control of target gene expression, such as the tamoxifen-regulated Cre-*loxP* and the tetracycline (Tet) regulatory systems, are sometimes favored.

**Figure 1:**
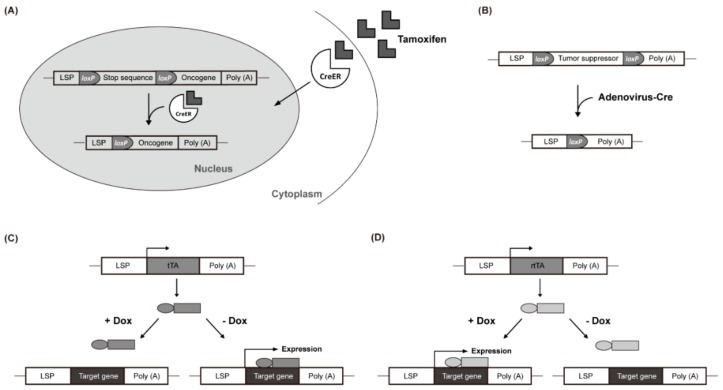
Liver-Specific Genetic Modification Models. (**A**) Cre-ER translocates into the nucleus and induces recombination between *loxP* sites. (**B**) recombinant adenovirus removes a floxed target gene. (**C**) doxycycline (Dox) suppresses the transcription of the gene of interest. (**D**) Dox activates rtTA by attaching it to the promoter and promotes the transcription of the target gene.

In the tamoxifen-regulated Cre-*loxP* system, the expression of a gene of interest can be induced with tamoxifen treatment. In the absence of tamoxifen, a Cre-estrogen receptor fusion protein (Cre-ER) remains in the cytosol. However, in response to tamoxifen introduced via an intraperitoneal injection, Cre-ER is translocated into the nucleus and induces recombination between *loxP* sites, resulting in the removal of transcriptional stop DNA elements (Figure 1A). This allows the target gene to be expressed in the liver by a liver-specific promoter (LSP) [69,70,71]. Virus-mediated Cre delivery systems are another option for temporal control of target gene expression in the liver. For example, a recombinant adenovirus expressing the *Cre* gene can be used to remove a floxed target gene (Figure 1B) [72,73]. Colnot et al. created a transgenic mouse in which the tumor suppressor gene *Apc* was located between two *loxP* sites. Intravenously injected Cre-adenovirus induced the deletion of *Apc* in a liver-specific manner. After 8 months, 67% of the mice developed liver cancer and the signaling pathway of *β-catenin* was strongly upregulated in the *Apc*-inactivated HCC [65].

The Tet-Off system uses the tetracycline transactivator (tTA), whereas the Tet-On system uses the reverse tetracycline-controlled transactivator (rtTA). In the Tet-Off system, doxycycline (Dox) suppresses the transcription of the gene of interest by preventing tTA from attaching to the promoter (Figure 1C). Conversely, in the Tet-On system, Dox activates rtTA by attaching it to the promoter, which, in turn, promotes the transcription of the target gene (Figure 1D). These inducible gene expression systems have been used in *c-Myc*-induced liver cancer studies. Shachaf et al. developed a mouse model in which *c-Myc* was regulated by the tetracycline promoter and tTA was regulated by the liver-specific LAP promoter [55]. The mouse model expressed *c-Myc* in the liver but not when treated with Dox (Tet-Off *c-Myc* model). Liver cancer occurred in all transgenic mice with upregulated *c-Myc* after approximately 12 weeks. After 4 days of Dox treatment, liver cancer differentiated into normal hepatocytes with apoptosis and almost all of it was eliminated within 2 weeks.

### 3.5. Hydrodynamics-Based Transfection and Sleeping Beauty (SB) Transposon

**Figure 2:**
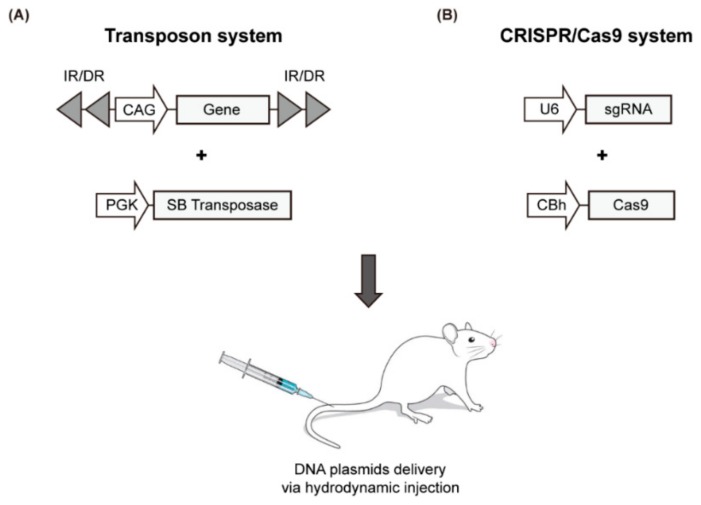
Schematic illustration of the HT-based mouse model using the SB transposase system (**A**) and CRISPR/Cas9 genome editing tool (**B**).

Recently, a simple liver-specific transgenic approach that employs the SB transposase system and the hydrodynamics-based transfection (HT) method was developed to create a mouse model for liver cancer (Figure 2A, Table 4). HT is a simple physical method to deliver naked DNA plasmids to liver cells [74]. As episomal plasmids only allow the transient expression of a target gene, chromosomal integration is required to sustain gene expression. The SB transposase mediates chromosomal integration of transposons; thus, the gene to be expressed is placed in the plasmid between specific repeating sequences (IRs). The transposase recognizes this position and integrates the gene into the chromosome [75,76,77]. A variety of transgenic mouse models have been developed using this method, through which the oncogenic signaling pathway is activated or the tumor suppressor pathway is inactivated. A selection of representative models using HT is summarized in Table 4 with emphasis on the most recent findings.

#### 3.5.1. Combination of Genetic Modifications and Disease-Specific Injury

Transgenic mice expressing an activated form of the *Akt* proto-oncogene, which were created using HT and the SB transposon, developed HCC after 6 months [78]. This model showed high similarity to a transgenic mouse model in which Pten was knocked out in the liver via traditional genetic manipulation [79].

Studies showed that transgenic mouse models overexpressing only the c-*Myc* proto-oncogene develop cancer in 60% to 70% of cases [80,81]. The tumors of c-*Myc*-induced models display high heterogeneity and mimic alcohol-induced HCCs, based on genomic changes. Additionally, a mouse model expressing *c-Myc* in the liver and developed using HT showed liver cancer in 5 to 8 weeks, exhibiting histological features similar to those seen in human hepatoblastoma [82]. Based on these findings, Chung et al. used a chemical induction methodology (CCl_4_) combined with HT to express c-Myc and a short hairpin RNA downregulating p53 expression (sh*p53*) in mice [83]. The tumor incidence with liver fibrosis was significantly higher in transgenic mice treated with CCl_4_ than in those treated with vehicle.

Recent studies also showed that the overexpression of more than two oncogenes or the combination of an activated oncogene and an inactivated tumor suppressor gene could effectively contribute in the development of HCC models to obtain shorter latency and increased tumor induction. For instance, activated *Met* and the mutation of the *β-catenin* gene are considered to be more frequently present in human HCCs [84,85]. To investigate their cooperation in tumorigenesis, Tao et al. co-expressed *Met* and *β-catenin* point mutants (S33Y or S45Y) in hepatocytes, leading to HCC development as early as 7 weeks after HT [86]. Additionally, the hepatic expression of active *β-catenin* and *Yap1* using HT and the SB transposon resulted in the development of hepatoblastoma (HB) in mice [87]. *SPRY2*, a gene with reduced function in half of human HCCs, is known to act as a tumor suppressor in the development of liver cancer via inhibition of the Ras signaling pathway [88]. The potential tumor suppressive role of *Spry2* in HCC was investigated by expressing a dominant negative form of Spry2 (*Spry2*Y55F) and an activated *β-catenin* in mouse liver through hydrodynamic injection and the SB transposon system.

The HT method coupled with the SB transposon system could be useful in studies of specific signaling roles in tumorigenesis. HCC mouse models were developed via hydrodynamic delivery of activated Ras combined with either *Tp53* downregulation or Taz activation. In these models, TGF-β signaling played a pro-tumorigenic role during the early stages of hepatocarcinogenesis, upregulating *Snai1* [89]. In addition, Wang et al. generated intrahepatic cholangiocarcinoma (ICCA) mouse models using this system. The overexpression of *Fbxw7*ΔF, a dominant negative form of *Fbxw7*, did not cause significant abnormalities in the mouse liver, whereas its co-expression with an activated form of Akt resulted in ICCA [90].

#### 3.5.2. The HT Model as a Tool for Preclinical Treatment

HCC models developed using HT and transposon methods are also very useful for evaluating the efficacy of anticancer drugs in the liver. Zhang et al. evaluated the therapeutic potential of mTOR inhibitor MLN0128 vs. gemcitabine/oxaliplatin using ICCA mouse models containing activated forms of Akt (*myr-Akt*) and Yap (*Yap*^S127A^). The study showed the antitumor potential of MLN0128 in ICCA, suggesting that it may be superior to gemcitabine/oxaliplatin-based chemotherapy, especially in tumors exhibiting activated Akt/mTOR signaling [91].

Liu et al. established an HCC preclinical mouse model co-expressing *Akt* and *c-Met* proto-oncogenes. In this model, the therapeutic efficacies of sorafenib, regorafenib, the MEK inhibitor PD901, and the pan-*mTOR* inhibitor MLN0128 were investigated. Treatment with PD901 or MLN0128 alone suppressed HCC growth. Simultaneous administration of both drugs showed a stronger inhibition effect on cell cycle and tumor growth. Thus, the combination of MEK and mTOR inhibitors may represent an effective therapeutic approach in human HCCs [92].

HT and transposon-based HCC models can also be used for testing a potential anti-HCC drug. Chen et al. identified niclosamide ethanolamine (NEN) as a potential antitumor agent through a bioinformatics-based search. In an HCC mouse model produced by hydrodynamic delivery of transposons expressing activated forms of Ras and β-catenin (N90), the oral administration of NEN significantly reduced HCC growth, with a more potent anticancer effect when combined with sorafenib [93].

The HT method coupled with the SB transposon system is an ideal approach to study the roles of diverse novel genes in hepatocarcinogenesis due to the simplicity and cost-effectiveness of these techniques. The HT and SB transposon method is expected to provide insights into the biology of hepatic tumorigenesis and allow us to test novel therapies towards precision medicine.

### 3.6. HT and CRISPR/Cas9

Murine models developed via HT have been widely used for genetic studies of liver cancer. Although the transgenic methodology has considerable advantages over the traditional GEM approach, which requires a series of time-consuming and resource-demanding steps, the transposon-based liver cancer models also present disadvantages. First, transposons encoding oncogenes are randomly integrated into chromosomes, affecting endogenous genes at or near the integration sites. This raises the possibility of tumorigenesis induced by oncogenes influenced by the genomic integration location. Second, the oncogenes in transposons are generally placed under the control of an ectopic and strong promoter; thus, genes are expressed at an extremely high level. These shortcomings might under or overrepresent the true tumorigenic potential of an oncogene at the endogenous locus.

To overcome these limitations, direct manipulation of endogenous genes in the liver has been attempted using the CRISPR/Cas9 genome editing tool, which has been successfully applied in many organisms. This genome editing technique utilizes an RNA-guided DNA endonuclease (Cas9 nuclease) and a single guide RNA (sgRNA) that guides the Cas9 to a complementary DNA sequence where it cleaves both DNA strands. During repair processes of double-stranded DNA breaks generated by Cas9, insertions and deletions are created (via non-homologous end joining) or a specific nucleotide sequence is introduced at the target site (via homology-directed repair). To induce genome editing by the CRISPR/Cas system in the liver, plasmids encoding Cas9 and sgRNA are delivered to the liver via the HT method (Figure 2B and Table 4).

The first successful application of the CRISPR/Cas9 system in a liver cancer model was reported in 2014 by Tyler Jacks’ research group [94]. Using HT for the hepatic delivery of Cas9 and sgRNAs targeting *Pten* and *Tp53*, they successfully and simultaneously induced somatic gene disruption in the murine liver tumor suppressor genes, in a manner similar to liver tumors induced by *Pten* and *Tp53* double knock-outs built via the traditional GEM approach. In a similar study, Liu et al. used HT and the CRISPR/Cas9 system to inactivate *Tp53* and *Pten* simultaneously in the livers of adult transgenic mice that expressed the HBV large envelope polypeptide [40,95]. The hepatic gene disruption of *Tp53* and *Pten* in the HBV transgenic mice significantly accelerated tumorigenesis in the liver, resulting in tumors as early as 4 months after HT.

Recently, Engelholm et al. employed HT and the CRISPR/Cas9 system to investigate the genetic aspects of the initiation of fibrolamellar hepatocellular carcinoma (FL-HCC), a liver cancer that predominantly affects children [96]. A 400 kb deletion on chromosome 19, which leads to gene fusion between DnaJ heat shock protein family member B1 gene (*DNAJB1*) and the protein kinase cAMP-activated catalytic subunit alpha gene (*PRKACA*), is repeatedly found in FL-HCC patients. Therefore, this study attempted to investigate the role of this gene fusion event in FL-HCC using HT and the CRISPR/Cas9 system. They designed sgRNA targeting intron 1 of murine *Dnajb1* and intron 1 of murine *Prkaca* to induce DNA double-strand cuts in these regions. When the sgRNA and Cas9 were delivered to wild-type mice via HT, DNA double-strand breaks were introduced at the two genetic loci as expected; the subsequent cellular repair led to DNA end joining that created the *Dnajb1–Prkaca* gene fusion, as observed in human FL-HCC. They found that mice with this gene fusion in the liver developed tumors that have many features of human FL-HCC.

Thus, the HT and the CRISPR/Cas9 system can be used to faithfully mimic the genetic features found in patients with liver cancer and applied to investigate the roles of genetic alterations in liver carcinogenesis. However, CRISPR/Cas9 technology should be applied on a liver fibrosis or cirrhosis background to correctly mirror human HCC.

## 4. Conclusions

A successful experimental model for HCC should reflect the biological characteristics of human HCCs; it should also be reliable, highly reproducible, and technically simple. To date, GEM models for liver cancer have provided invaluable information on HCC, such as the function of oncogenes and tumor suppressor genes, the interaction between tumor and host cells, cellular responses to chemotherapeutic agents, and the role of stem cells in tumor progression. Traditional GEM methodology requires a series of time-consuming and resource-demanding procedures that delay the development of a variety of liver cancer models. HT methodology, combined with either the SB transposon system or the CRISPR/Cas9 technique, is a promising alternative to the traditional approach for generating GEM models for liver cancer (Table 5). Further development and refinement of the HT-based GEM models are expected to broaden our knowledge of the genetic mechanisms underlying hepatocarcinogenesis and provide a novel therapeutic strategy targeting genes that maintain and promote liver cancer.

## Figures and Tables

**Table 1:** Chemically induced models.

Diet or Chemical	Mechanism of Action	Phenotype	Dose & Route	References
Diethylnitrosamine (DENA)	Genotoxic hepatocarcinogen	50–90 weeks: 100% HCC	75–100 mg/kg IP	[7,8,9]
N-nitrosomorpholine (NMOR)	Genotoxic	12 weeks: HCC with lung metastasis	120 ppm w/drinking water	[10]
Choline-deficient and ethionine (CDE) diet	Oxidative DNA damage, DNA strand breaks, and chromosomal instability	30–35 weeks: 100% HCC	Feeding	[11,12,13]
2-Acetylaminofluorene (2-AAF)	Genotoxic	Used primarily as promoter in initiation/ promotion protocols	10 mg/kg Oral administration	[14,15]

HCC, hepatocellular carcinoma; IP, Intraperitoneal injection.

**Table 2:** Transplantation models.

Type of Sample	Characteristics (Anatomical Location)	Advantage	Disadvantage
Ectopic tumor xenograft model (subcutaneous model)	Different origin from the cultured cells	Easy monitoring of tumorigenicity and tumor growth	-Unable to mount an immune response -Unable to predict response to antitumor agents
Orthotopic model	implanted into the equivalent organ from which the cancer originated	Reproducing the histology of human tumors, local invasion, and ex vivo genetic manipulation	-Unable to mount an immune response -Unable to replicate early oncogenesis
Syngeneic model (allograft mouse model)	Tumor tissues derived from the same genetic background as a given mouse strain	Intact retention of the immune system, which is beneficial for immunotherapy studies	Differences between the mouse and human immune systems, need for mouse reactive agents
Patient-derived tumor xenograft model (PDTX)	Transplantation of the cancer patient tissue directly into immunocompromised mice	-Genetic, histological, and phenotypic similarities with the tumor -Predicting the response to anticancer drugs	-Expensive -Time-consuming

**Table 3:** HCC mouse models with proto-oncogenes or tumor suppressor genes.

Proto-Oncogene	Tumor Suppressor Gene	Time to Development	Characteristics
*c-Myc*/*TGF-α*		15 months	Increased ROS and genetic instability Loss of heterozygosity
*β-Catenin*/*RAS*		8 weeks	Well-differentiated HCC with a compact and trabecular pattern
*Ras* + *c-Myc*		2 months	Moderately differentiated HCC
*Ras*	*p53*	1 months	Poorly differentiated HCC
*c-Myc*	*p53*	7 months	Well-differentiated HCC
*myr-Akt* + *NRas*V12		3–4 weeks	Mixed HCC and ICCA
*myr-Akt* + *Spry2*Y55F		3–4 months	HCC with emperipolesis
*NICD1*		4–5 months	ICCA
*myr-Akt* + *NICD*		–3 weeks	ICCA

ICCA, intrahepatic cholangiocarcinoma; ROS, reactive oxygen species; HCC, hepatocellular carcinoma; ICCA, intrahepatic cholangiocarcinoma.

**Table 4:** Mouse models of HCC generated via hydrodynamics-based transfection.

Modulation System	Target Genes	Tumor Type	Mouse Strain	Latency	References
Sleeping Beauty transposon	*c-Myc*	HB	WT FVB/N	~6 weeks	[82]
	*Spry2*Y55F + ΔN90 *β-catenin*	HCC	WT FVB/N	~6 months	[88]
	*hMet* + *β-catenin* (*S33Y* or *S45Y*)	HCC	WT FVB/N	~6 weeks	[86]
	*c-Myc* + sh*P53*	HCC	WT C57BL/6	~7 weeks	[83]
	*YAP*S127A + ΔN90 *β-catenin*	HB	WT FVB/N	~6 weeks	[91]
	*myr-Akt* + *Fbxw7*ΔF	ICCA	WT FVB/N	~6 weeks	[90]
	*Smad7* + *HRAS*G12V + sh*P53*	HCC	WT C57BL/6	~5 weeks	[89]
	*myr-Akt* + *YAP*S127A	ICCA	WT FVB/N	~3 weeks	[91]
	*myr-Akt* + *cMet*	HCC	WT FVB/N	~4 weeks	[92]
	*Ras*V12 + ΔN90 *β-catenin*	HCC	WT FVB/N	~4 weeks	[93]
CRISPR/Cas9	sg*Pten* + sg*P53* + Cas9	CK19-positive liver tumors	WT FVB/N	~3 months	[95]
	sg*Dnajb1* + sg*Prkaca* + Cas9	FL-HCC	WT FVB/N	~14 months	[96]

HB, hepatoblastoma; FL-HCC, fibrolamellar hepatocellular carcinoma.

**Table 5:** Methodologies for creating genetically engineered mouse models for liver cancer.

Method	Advantages	Shortcomings
Traditional transgenic and knock-out techniques	Modification of endogenous gene (knock-in and knock-out) No genetic variation in an established line	Technically challenging Resource-demanding Subsequent breeding and animal maintenance required 1–2 years to establish a model
Hydrodynamics-based transfection and Sleeping Beauty transposon	Simple and easy procedure A few weeks to establish a model	Random integration of transgenes Usually uses an ectopic promoter Genetic variation within a model (e.g., transgene copy, integration site)
Hydrodynamics-based transfection and CRISPR/Cas9	Simple and easy procedure A few weeks or months to establish a model Modification of endogenous genes	Possible genetic variation within a model (e.g., off-target genome editing, sequence variation at the target site)

Hepatocellular carcinoma, HCC; hepatoblastoma, HB; intrahepatic cholangiocarcinoma, ICCA; genetically engineered mouse, GEM; hydrodynamics-based transfection, HT; hepatitis B virus, HBV; hepatitis C virus, HCV; woodchuck hepatitis virus, WHV; tumor microenvironment, TME; liver activator protein, LAP; reactive oxygen species, ROS; tetracycline, Tet; cre-estrogen receptor, Cre-ER; liver-specific promoter, LSP; doxycycline, Dox; Sleeping Beauty, SB; single guide RNA, sgRNA; adenomatous polyposis coli, APC; hydrodynamics-based transfection, HT; niclosamide ethanolamine, NEN.
